# Voice quality and speech fluency distinguish individuals with Mild Cognitive Impairment from Healthy Controls

**DOI:** 10.1371/journal.pone.0236009

**Published:** 2020-07-13

**Authors:** Charalambos Themistocleous, Marie Eckerström, Dimitrios Kokkinakis

**Affiliations:** 1 Department of Neurology, Johns Hopkins University, Baltimore, Maryland, United States of America; 2 Department of Psychiatry and Neurochemistry, University of Gothenburg, Gothenburg, Sweden; 3 Department of Swedish, University of Gothenburg, Gothenburg, Sweden; 4 Center of Ageing and Health—AgeCap, University of Gothenburg, Gothenburg, Sweden; Nathan S Kline Institute, UNITED STATES

## Abstract

Mild Cognitive Impairment (MCI) is a syndrome characterized by cognitive decline greater than expected for an individual's age and education level. This study aims to determine whether voice quality and speech fluency distinguish patients with MCI from healthy individuals to improve diagnosis of patients with MCI. We analyzed recordings of the Cookie Theft picture description task produced by 26 patients with MCI and 29 healthy controls from Sweden and calculated measures of voice quality and speech fluency. The results show that patients with MCI differ significantly from HC with respect to acoustic aspects of voice quality, namely H1-A3, cepstral peak prominence, center of gravity, and shimmer; and speech fluency, namely articulation rate and averaged speaking time. The method proposed along with the obtainability of connected speech productions can enable quick and easy analysis of speech fluency and voice quality, providing accessible and objective diagnostic markers of patients with MCI.

## Introduction

Mild Cognitive Impairment (MCI) is a syndrome characterized by cognitive decline greater than expected for an individual's age and education level. Patients with MCI remain functional in their daily activities [1]. Progression rates vary across studies depending on the diagnostic criteria and methods being employed, although there are indications that about 50% of patients with MCI progress to Alzheimer’s Disease (AD) within five years, yet many patients remain stable for several years [1–3]. Currently, there is no cure for AD, but identifying patients with MCI early and applying therapy in a timely manner can delay the progression of the MCI to AD [4]. It is of utmost importance, to develop straightforward, not intrusive, and reliable objective diagnostic measurements of cognitive impairment that can be conducted at primary care centers and memory clinics to determine whether an individual should seek further professional advice.

Speech can provide such objective measures for the identification of patients with MCI. As language impairment is a common symptom of AD, affecting most language domains and functions including phonetics [5, 6], phonology [7], morphosyntactic structure (e.g., mean length of utterances, proportions of nouns and verbs, and syntactic complexity measures), semantics [8, 9], discourse and conversation [7, 10, 11], it can be employed to provide objective diagnostic markers. One of the less understood and studied aspects of language is speech production in patients with MCI [12, 13] and more research is required on the speech of patients with MCI as speech can convey information about the underlying language system and how it interacts with other language domains [14–16]. For instance, the slow recall of words can affect speech fluency, especially durational and frequency measures, tonal modulation, and pauses [14–18]. Speech can convey information about motoric and cognitive abilities of patients with MCI that relate to articulation, voice quality, and fluency. König et al. [12] employed automated acoustic measures and classified patients with MCI and healthy controls (HC) with 79% classification accuracy, patients with MCI and AD with 80% accuracy, and patients with AD from HC with 89% classification accuracy. In our previous work [19], we analyzed segmental and prosodic features of speech production and showed that vowel formants (F1 to F5), the fundamental frequency, and vowel duration can distinguish patients with MCI from HC with 83% mean cross-validated accuracy.

This study aims to identify a few selected features from voice quality and speech fluency that can function as objective markers distinguishing patients with MCI from HC. An advantage of this study over other studies of language in MCI is that our approach does not require preprocessing, such as transcription and segmentation of the acoustic signal into vowels and consonants. Specifically, we tested two main questions: i. Does voice quality (as estimated by differences between speech harmonics and amplitudes, the cepstral peak prominence (CPP), mean energy concentration of spectral or first spectral moment, the Hammarberg index, jittering, and shimmer) distinguish patients with MCI from HC? And ii. Do measures of speech fluency (namely, the averaged speaking time, articulation rate, and speech rate) distinguish patients with MCI from HC? To answer these questions, we are providing an acoustic analysis of speech productions from the Cookie Theft picture description task from the Boston Diagnostic Aphasia Examination (BDAE) produced by Swedish patients with MCI and HC [20]. This study shows that voice quality and speech fluency provide information that can identify patients with MCI from HC.

## Patients and methods

### Participants

The 55 participants were recruited as part of the Gothenburg MCI study, which is a large clinically based longitudinal study on MCI [21]. Details about the participants are provided in Table 1. The Gothenburg MCI study provides an in-depth phenotyping of patients with different forms and degrees of cognitive impairment using imaging/physiologic methods, psychometrics, and biochemical methods, namely cerebrospinal fluid characterization of substances in the brain. Participants were selected based on specific inclusion and exclusion criteria: (i) no dyslexia and other reading deficiencies; (ii) no current history of major depression, and recent substance abuse; (iii) no history of serious psychiatric, neurological and other brain-related conditions; (iv) to be native Swedish speakers; (v) to be able to read and understand information about the study; and (vi) to be able to provide written consent. HC had a significantly higher Mini-Mental State Exam score (*M* = 29.6). The MMSE score is a scale of 0–30 and represents the cognitive status of an individual. Mean MMSE score for the MCI participants was 28.2, which is close to normal [22–24]. Ethic approvals, the consent procedure, and data acquisition were approved by the Swedish Ethical Review Authority, <http://www.epn.se/> (ref. nr: 206–16, 2016) and the ethics amendment was approved by the same institution (ref. nr: T021-18, 2018). Subjects were prospectively recruited from one center: the Memory Clinic at the Sahlgrenska University Hospital, Sweden. All patients provided written informed consent for use of data before the data collection.

**Table 1:** Demographic information and scores for memory & learning, language, attention, and executive function, by group (mean and standard deviation).

		HC (n = 29)	MCI (n = 26)	Sig.
Demographic	Age (years)	67.8 (7.7)	70.6 (5.8)	*
	Education (years)	13.3 (3.7)	14.3 (3.6)	n.s.
	Sex (F/M)	21/8	14/12	n.s.
	MMSE (/30)	29.6 (0.6)	28.2 (1.4)	***
Memory/Learning	RAVLT (total)	45.5 (11.1)	37.6 (10.7)	*
	RAVLT (immediate)	9.5 (3.5)	6.1 (3.1)	***
	RAVLT (delayed)	9.2 (3.6)	5.8 (3.5)	***
	RCF (3 min)	18.8 (5.1)	15.8 (6.8)	n.s.
	RCF (20 min)	18.6 (4.4)	14.3 (7.0)	*
	WLM (immediate)	25.8 (6.3)	21.3 (7.6)	*
	WLM (delayed)	21.9 (8.1)	16.0 (10.5)	*
Language	BNT	53.3 (4.6)	50.2 (7.6)	n.s.
	Verbal Fluency (F-A-S)	47.2 (11.5)	43.6 (11.1)	n.s.
	Similarities (WAIS-III)	24.6 (4.7)	24.0 (5.2)	n.s.
	Token Test (Part 5)	20.9 (1.4)	20.0 (1.8)	n.s.
Attention	Digit Span (WAIS-III)	13.1 (3.5)	12.4 (2.8)	n.s.
	Digit-Symbol (WAIS-R)	62.9 (12.3)	54.2 (10.8)	**
	TMT A	34.1 (11.9)	39.5 (13.3)	n.s.
	TMT B	79.8 (32.9)	97.8 (49.4)	n.s.
	Block design (WAIS-III)	40.6 (9.5)	35.5 (12.2)	n.s.
	RCF (copy)	33.6 (2.4)	32.4 (3.4)	n.s.
	Silhouettes (VOSP battery)	22.4 (4.2)	19.3 (3.3)	***
Executive Function	Letter-number sequencing (WAIS-III)	9.5 (2.3)	8.7 (2.6)	n.s.
	PaSMO	68.2 (21.5)	86.8 (29.1)	*
	Stroop (Victoria version) (trial 1)	13.2(2.4)	14.6 (3.1)	n.s.
	Stroop (trial 2)	17.6(3.4)	19.4 (5.4)	n.s.
	Stroop (trial 3)	24.1(6.6)	27.6 (6.6)	*
	Stroop Effect	1.8(0.4)	1.9 (0.5)	n.s.

BNT Boston Naming Test [25]; PaSMO Parallel Serial Mental Operation (a measure of mental control and working memory where the subject is asked to recite the alphabet, stating the number after each letter, i.e., A-1-B-2-C-3…) [26]; RAVLT Rey Auditory Verbal Learning Test [27]; RFC Rey–Osterrieth complex figure (RCF) [28]; TMT A, TMT B Trail Making Test A and B [29]; VOSP Visual object and space perception battery [30]; WAIS Wechsler adult intelligence scale [31]; WLM: Wechsler logical memory [31]

‘*’ p < .05

‘**’ p < .01

‘***’ p < .001.

### Procedure and acoustic measurements

MCI diagnosis was based on staging of cognitive and functional abilities using the Geriatric deterioration scale (GDS) (GDS stage 3 = MCI) [32]. Specific operationalization of the GDS scoring in the Gothenburg MCI study has been described previously in detail [21]. The MCI group was mixed—we did not categorize the patients into MCI subgroups (such as amnestic MCI and non-amnestic MCI). A physician and/or registered nurse conducted the GDS assessment procedure, and the neuropsychological tests were administered by licensed psychologists alternatively health care professionals supervised by a licensed psychologist. Neuropsychological tests were selected by specialized psychologists, comprising tests within the cognitive domains speed and attention, learning and episodic memory, visuospatial, language, and executive functions. Testing was performed during clinical visits.

The picture description task was part of additional assessment tests conducted as part of “Linguistic and extra-linguistic parameters for early detection of cognitive impairment” research project funded by Riksbankens Jubileumsfond–The Swedish Foundation for Humanities & Social Sciences (NHS 14–1761:1). This picture shows two children trying to remove cookies from a jar placed on top of a cupboard as their mother is washing the dishes. A speech and language pathologist presented the picture to participants and prompted them to tell everything they see on the picture following the standard BDAE version 3 instructions. The picture description task was audio recorded using a Zoom H4N audio recorder, located at a fixed distance (1ft) in front of the participant. The audio was subsequently converted to 16000 Hz mono format [19, 33] and analyzed acoustically using the open source software for acoustic analysis Praat [34]. Specifically, we analyzed speech sounds and measured acoustic properties related to voice quality and speech fluency. Measurements of voice quality and syllable structure were calculated.

#### 1. Voice quality / phonation

Phonation and voice quality account for the fine control of the sublaryngeal and laryngeal systems. To determine the phonation and voice quality differences of patients with MCI and HC, we have calculated the following measurements.

- *H1-H2*, *H1-A1*, *H1-A3*: Difference between the amplitude of the first and second harmonics (H1-H2), the amplitude of the first harmonic and the amplitude of strongest harmonic of the first formant frequency (H1-A1), and the amplitude of the first harmonic and the amplitude of the third formant (H1-A3) demarcate voice quality. Harmonics are estimated by considering the fundamental frequency, and amplitudes from the spectra. H1-H2 indicates breathy (strong H1) and creaky voice (weaker H2) [35].
- *Cepstral Peak Prominence (CPP)*: CPP is a reliable measure of dysphonia [36]. It accounts for the periodicity in the voice signal: higher values of CPP correspond to greater periodicity. It stands as the relative amplitude of the CPP in relation to the expected amplitude as derived via linear regression.
- *Mean Energy Concentration*: or *first spectral moment* is the average spectral frequency [37, 38].
- *Hammarberg Index*: The Hammarberg index is the difference between the maximum energy in the 0…2kHz energy band and the energy in the 2…5kHz band. The Hammarberg index is considered an indicator of articulatory effort [39].
  Finally, we provide measures of shimmering, jittering, and harmonicity elicited using Praat [34].
- *Jitter* (Hz): it is the cycle-to-cycle variation of the *fundamental frequency* (*F*_0_) (1), expressed as:
  $$Jitter=\frac{1}{N-1}\sum_{i=1}^{N-1}\left|T_{i}-T_{i+1}\right|$$ (1)
  where *T*_*i*_ are the extracted period lengths and *N* is the number of extracted *F*_0_ periods. The *F*_0_ is the basic frequency produced during the vibration of the vocal folds and it is one of the primary acoustic correlates of intonation, which manifests linguistic (e.g., different melodic patterns for questions, and statements) and extralinguistic functions (e.g., emotional prosody) [40]. Reduced control on vocal-fold vibration results in higher percentage of jitter [41].
- *Shimmer* (dB): it is the variability of the amplitude from peak-to-peak (local maxima). Eq (2) shows shimmer as the mean absolute base-10 logarithm (multiplied by 20) of the difference between the amplitudes of successive periods (2):
  $$Shimmer\;(dB)=\frac{1}{N-1}\sum_{i=1}^{N-1}\left|20\;\log\left(\frac{A_{i+1}}{A_{i}}\right)\right|$$ (2)
  where *A*_*i*_ are the extracted peak-to-peak measurements of amplitude and *N* is the number of *F*_0_ periods. Shimmer indicates noisy productions and breathiness and it is a correlate of glottal resistance and mass lesions on the vocal folds [41].

#### 2. Speech fluency

*Speech rate and articulation rate*. These are measures of fluency as described in the introduction. We calculated the following measures: average syllable duration, the articulation rate, and speech rate.

- *Average Syllable Duration*: Is the mean syllable duration estimated as a measure of the overall speaking time divided by the number of syllables (3).
  $$Averaged\;Syllable\;Duration=\frac{Overall\;Speaking\;Time}{Number\;of\;Syllables}$$ (3)
- *Articulation Rate*: Articulation rate considers phonation time, which is a measure of phonation times and excludes pauses and silences (4).
  $$Articulation\;rate=\frac{Number\;of\;Syllables}{phonation\;time}$$ (4)
- *Speech Rate*: Is a measure of the number of syllables divided by the overall duration, which includes pauses and silences (5):
  $$Speech\;rate=\frac{Number\;of\;Syllables}{Total\;Duration}$$ (5)

For the statistical analysis, we employed linear mixed effects models using condition (MCI vs. HC) and gender as fixed factors on voice quality and phonation measurements (dependent variables) and condition on speech fluency measurements dependent variables. We included gender in the statistics of voice quality and phonation, as these measures depend on physiological differences between men and women, e.g., lower pitch in men than in women. The *R* package *emmeans* was employed to obtain estimated marginal means (EMMs, also known as least-squares means) for factor combinations in the linear mixed effects models and compute the contrasts or linear combinations of these marginal means.

## Results

### Voice quality / phonation

**Fig 1:**
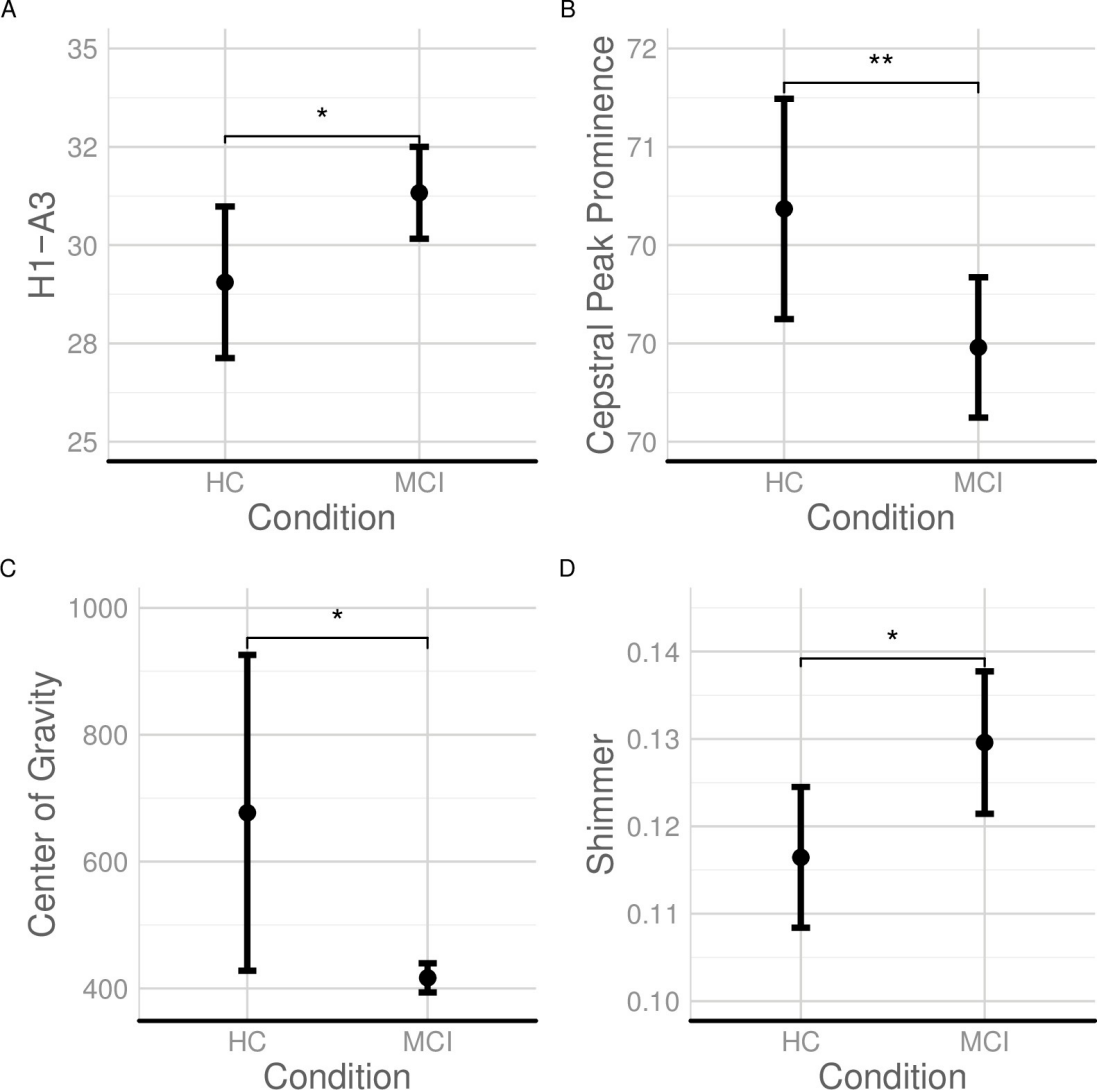
Voice quality and phonation elicited from audio recordings produced by patients with MCI and HC. (A) H1-A3 (dB); (B) Cepstral Peak Prominence (dB); (C) Center of Gravity (Hz); and (D) Shimmer (dB) in HC and patients with MCI; ‘.’ indicates p < 0.1; ‘*’ indicates p < .05; ‘**’ indicates p < .01.

Voice quality measures demonstrate significant differences between patients with MCI and HC as shown in Fig 1. Patients with MCI produce speech that differs from HC in phonation and voice quality, which is measured using objective markers presented in this section and determine differences in the fine-control of the sublaryngeal and laryngeal systems. We found significant differences of patients with MCI from HC with respect to the difference of the first harmonic and third amplitude (H1-A3), shown in Table 2. Patients with MCI differed significantly from HC with respect to their CPP (see Fig 1, Panel B). There is an overall lower CPP in patients with MCI compared to HC, suggesting weaker voice. Also, patients with MCI differed significantly from HC with respect to shimmer and center of gravity. However, patients with MCI and HC did not differ significantly with respect to the Hammarberg Index measurement (*F*(1:278) = 0.137, *p* = 0.711). Also, there were no significant differences between patients with MCI and HC in jitter (*F* (1, 254) = 2.73, *p* = 0.1).

**Table 2:** Regression results for the effect of condition (MCI vs. HC) and gender on H1-A3.

	Predictor	*b*	*b*	*sr*^*2*^	*sr*^*2*^	Fit
			95% CI		95% CI	
			[LL, UL]		[LL, UL]	
*H1-A3*	Intercept	26.87**	[25.14, 28.59]			*R*^*2*^ = .137**
	MCI	2.91*	[0.52, 5.29]	.02	[-.01, .05]	95% CI[.06,.21]
	Male	9.75**	[6.11, 13.39]	.09	[.03, .15]	
	MCI:Male	-5.52*	[-10.06, -0.97]	.02	[-.01, .05]	
CPP	Intercept	71.12**	[70.59, 71.66]			*R*^*2*^ = .057**
	MCI	-1.18**	[-1.92, -0.44]	.03	[-.01, .07]	95% CI[.01,.11]
	Male	-1.96**	[-3.08, -0.83]	.04	[-.00, .08]	
	MCI:Male	2.05**	[0.64, 3.46]	.03	[-.01, .07]	Center of Gravity
Center of Gravity	Intercept	676.96**	[513.71, 840.22]			*R*^*2*^ = .020*
	MCI	-260.32*	[-476.29, -44.35]	.02	[.00, .06]	95% CI[.00,.06]
Shimmer	Intercept	0.12**	[0.11, 0.13]			*R*^*2*^ = .019*
	MCI	0.01*	[0.00, 0.02]	.02	[.00, .06]	95% CI [.00,.06]

*b* unstandardized regression weights, a significant *b*-weight indicates the semi-partial correlation is also significant.; *sr*^*2*^ semi-partial correlation squared; *LL* and *UL* lower and upper limits of a confidence interval

* *p* < .05

** *p* < .01.

### C2. Speech fluency

**Fig 2:**
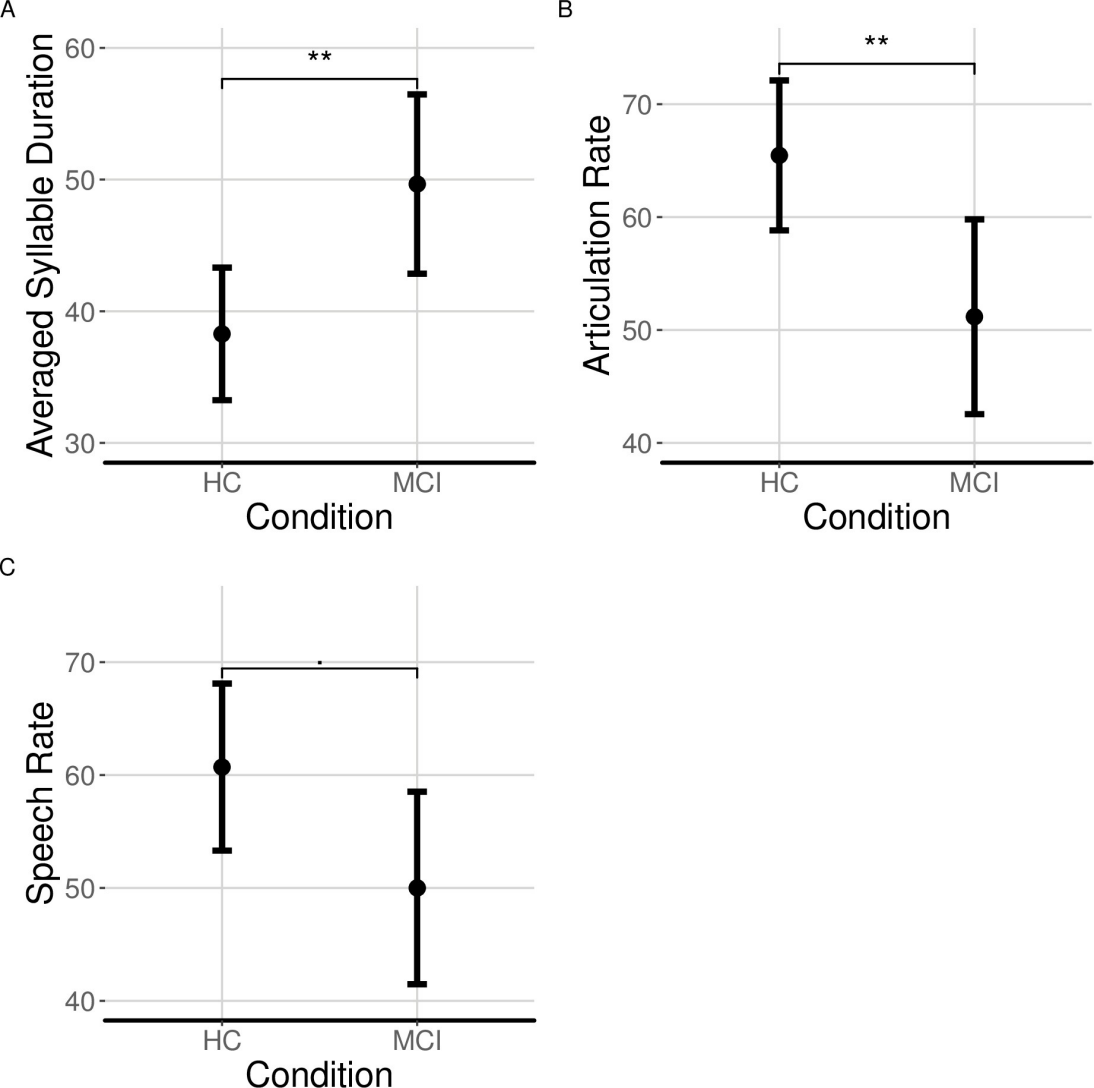
Measures of speech fluency elicited from audio recordings produced by patients with MCI and HC. (A) averaged speaking time; (B) articulation rate; and (C) speech rate in HC and patients with MCI; ‘.’ indicates p < 0.1; ‘*’ indicates p < .05; ‘**’ indicates p < .01.

Patients with MCI produced significantly longer syllables from HC, as measured by the average syllable duration and had a slower articulation rate and speech rate but only with respect to average syllable duration and articulation rate we found significant effects (see Fig 2 and Table 3).

**Table 3:** Regression results for the effect of Condition (MCI vs. HC) on averaged syllable duration, articulation rate, and speech rate.

	Predictor	*b*	*b*	*sr*^*2*^	*sr*^*2*^	Fit
			95% CI [LL, UL]		95% CI [LL, UL]	
Average Syllable Duration	Intercept	38.28**	[32.86, 43.71]			*R*^*2*^ = .063**
	MCI	11.38**	[3.19, 19.57]	.06	[.00, .16]	95% CI[.00,.16]
A*rticulation Rate*	Intercept	65.47**	[58.46, 72.48]			*R*^*2*^ = .060**
	MCI	-14.29**	[-24.87, -3.71]	.06	[.00, .16]	95% CI[.00,.16]
*Speech Rate*	Intercept	60.70**	[53.32, 68.08]			
	MCI	-10.70	[-21.85, 0.44]	.03	[.00, .12]	
						*R*^*2*^ = .031
						95%CI[.00,.12]

*b* unstandardized regression weights, a significant *b*-weight indicates the semi-partial correlation is also significant; *sr*^*2*^ semi-partial correlation squared; *LL* and *UL* lower and upper limits of a confidence interval

‘*’ *p* < .05

‘**’ *p* < .01.

Table 4 presents a summary of the main findings with the acoustic measures that differentiate patients with MCI from HC.

**Table 4:** Summary of the main findings.

	Measure	Result
Voice Quality	H1-A3	Significant differences between patients with MCI vs HC
	Cepstral Peak Prominence	Significant differences between patients with MCI vs HC
	Center of Gravity	Significant differences between patients with MCI vs HC
	Shimmer	Significant differences between patients with MCI vs HC
Articulation Rate	Average Syllable Duration	Significant differences between patients with MCI vs HC
	Articulation Rate	Significant differences between patients with MCI vs HC
	Speech Rate	Marginal differences (p < 0.1).

## Discussion

Cognitive decline in patients with Mild Cognitive Impairment (MCI) is manifested as a noticeable memory difficulty in remembering events and situations, impaired language, speech, decision making, planning, interpreting instructions, and orientation [1, 21, 42–48]. Given that MCI patients are a high risk group for developing AD, there is a dire need to elicit objective measures that can enable the early and quick identification of patients with MCI, to provide treatment promptly, facilitate MCI prognosis, and ultimately improve life quality both for patients with MCI and for their family members. This study provides novel findings that show impairment of speech production in patients with MCI with respect to (i) voice quality and (ii) speech fluency and demonstrates that these measures can provide objective diagnostics of patients with MCI.

### Voice quality measures of MCI

An unexpected finding is that patients with MCI differed from HC with respect to voice quality. Early cognitive impairment is manifested by disparities in voice breathiness and increased dysphonia. Patients with MCI differed from HC in H1-A3, which suggests that voice breathiness is different in patients with MCI with respect to HC. Our study shows an increased H1-A3 in patients with MCI with respect to HC. Tanaka, Adachi [49] report a similar finding in patients with AD vs. HC. A novel finding was that patients with MCI show lower periodicity in spectra than HC, which corresponds to greater dysphonia, as measured with the CPP. Patients with MCI are characterized by overall lower center of gravity; which can correspond to lower frequency speech productions, that result into a significantly weaker speech than HC of the same age. It also indicates an overall relaxation of articulators during speech production that is manifested by the lowering of the spectral center of gravity. Patients with MCI are characterized by greater shimmer in speech production which indicates greater instability of amplitude. Greater shimmer may indicate less stability and control of the sublaryngeal/pulmonary pressure. Another important finding is that patients with MCI are characterized by differences in breathy voice, greater dysphonia, lower center of gravity and shimmer. These findings may be the result of cognitive and physiological impairment of the fine control and the slowing down of the vocal folds, of pulmonary pressure, respiration, and the co-ordination of phonation with articulatory production [15, 50–53].

### Speech fluency measures

Patients with MCI have different speech fluency measures. Our findings show that the overall articulation rate and speech rate are significantly slower in patients with MCI than in elderly HC. The slower articulation rate can be the result of slower cognitive processes due to MCI, affecting attention, memory, and language, including word recall and grammar [1–3]. It can also be the result of impaired motor control as patients with MCI are characterized largely by abnormalities in motor coordination and disinhibition [54], motor preparation [55], and motor planning [56], which can influence motoric functions related to articulation.

### Diagnostic utility of speech features

This study brought together speech acoustics and statistical analysis for the study of speech production in MCI. Speech reveals multidimensional information about the speaker (e.g., age, gender, sociolinguistic characteristics, physiological condition) and can function as a fingerprint that identifies patients with MCI from HC. The findings provide objective measures from voice quality that distinguish patients with MCI and HC and at the same time they point to the importance of phonation and speech fluency as a diagnostic measurement [50–53]. Implemented as a computer application, this approach can provide an easy and accessible interface for the automatic quantification of voice quality and speech fluency, utilized by physicians, neuropsychologists, and speech therapists to quantify speech in tasks, such as picture description tasks, scripts, and discourse. By increasing the span of acoustic measurements that can be analyzed and understanding their corresponding speech deficits [57, 58], physicians, neuropsychologists, and speech therapists can tailor therapeutic programs to the specific needs of their clients (e.g., focusing on targeted part of speech productions). Measures of voice quality and fluency from connected speech, discourse, etc. can enable clinicians to assess the overall speech production of patients with MCI and provide information about the differential speech properties of patients with MCI variants and HC and ultimately enable a better understanding of speech symptoms of patients with MCI.

### Limitations and future directions

Picture description tasks (e.g., Cookie Theft) constrain the production of speech in that the productions are often narrowed down to labelling rather than on free narration which to a certain degree may constrain fluency measurements. In contrast, storytelling, discourse, and conversation are characterized by expressive variations of fluency. This aspect of fluency cannot be tested using picture description tasks but requires a computational analysis of voice quality and speech fluency in free style conversations and in other conditions affecting language, such as stroke aphasia and primary progressive aphasia [e.g., 58, 59, 60–64]. Another limitation is the relatively small sample size; a larger sample size is expected to increase the effect size of the model. Also, as speakers are recruited at a single recruitment center, the participants may not be representative of the overall population of patients with MCI in Sweden. To address these limitations, we are collecting a variety of linguistic data from a larger population of patients attending different recruitment centers. The acoustic measures proposed in this study along with the obtainability of connected speech productions and the availability of acoustic analysis software can enable the rapid analysis of speech in the primary care centers and memory clinics providing accessible diagnostic methods for MCI.
